# Are congenital malformations associated with maternal sociodemographic and risk factors? A multicenter ultrasound-based study

**DOI:** 10.12669/pjms.40.11.9792

**Published:** 2024-12

**Authors:** Jawaria Aslam Awan, Aisha Qamar, Ambreen Surti, Eman Anwar

**Affiliations:** 1 Dr. Jawaria Aslam Awan, BDS, MPhil. Senior Lecturer - Department of Anatomy, Bahria University Health Sciences Campus Karachi (BUHSCK), Karachi, Pakistan; 2 Prof. Dr. Aisha Qamar, MBBS, MPhil. Senior Professor - Department of Anatomy, Bahria University Health Sciences Campus Karachi (BUHSCK), Karachi, Pakistan; 3 Dr. Ambreen Surti, MBBS, MPhil, MHPE. Assistant Professor, Department of Biological and Biomedical Sciences, Aga Khan University, Karachi, Pakistan; 4 Eman Anwar, 3^rd^ Year MBBS Student, Aga Khan University, Karachi, Pakistan

**Keywords:** Congenital Malformation, Abnormalities, Maternal, Sociodemographic Factors, Risk Factors

## Abstract

**Objective:**

Congenital malformations (CM) are structural defects of the human body that arise during development. They significantly impact neonatal outcomes such as morbidity and mortality, hence identification of risk factors and their reduction is vital to improve materno-fetal outcomes. In Karachi, Pakistan, there is insufficient data on the incidence of congenital malformations. Therefore, it is necessary to initiate a prospective case control study. The desired objective was to determine the association of congenital malformations with sociodemographic and maternal risk factors in different ethnicities.

**Methods:**

This multi-center case control study spanned over a duration of six months (December 2022 – May 2023) and included women ranging from 18–45 years using purposive sampling technique. It was conducted at Jinnah Postgraduate Medical Center and Tanveer Ultrasound Clinic. Sample of 120 participants fulfilling the inclusion criteria were included in this study after purposive sampling. Sonoscape S22 ultrasound system and Toshiba Aplio 500 ultrasound system were used to detect congenital malformations.

**Results:**

Participants were divided into five groups based on ethnicity. Most of the congenital anomalies of the central nervous system were seen in Urdu-speaking group with a p-value = 0.016. An association between maternal age, education level, family income and medications with congenital malformations in fetuses was observed with p-values of 0.003, 0.000, 0.024 and 0.000 respectively.

**Conclusion:**

It was evident that various maternal sociodemographic and risk factors play a significant role in the development of congenital anomalies.

## INTRODUCTION

Congenital malformations (CM) are structural defects that occur during intrauterine life. They significantly impact neonatal outcomes such as morbidity and mortality, hence identification of risk factors and their reduction is vital to improve maternofetal outcomes.^1^ In underdeveloped countries, there is lack of suitable health infrastructure to manage congenital malformations. Therefore, focusing on modifiable socioeconomic determinants to reduce congenital abnormalities may be more advantageous.^2^

The estimated prevalence of CM globally is 5%.^3^ In Pakistan it contributes to 6-9% of perinatal deaths.^4^ The etiology varies from maternal infections and morbidities to genetic causes.^3^ Maternal hypertension, Type-II diabetes and obesity have been observed to be associated with congenital malformations, especially congenital heart disease.^5^ Previous research has revealed that hypertensive disorders in pregnancy (HDP) cause an undesirable in utero environment, leading to low birth weight, neonatal mortality, intrauterine growth restriction, congenital abnormalities, and so on.^3^,^6^ However, many cases remain unexplained and no cause can be identified for them.^7^

Maternal nutritional status and lifestyle practices also play a role in the development of CM. Lack of folic acid supplementation, environmental factors, dietary deficiencies, and use of prescription drugs are some of the major risk factors for neural tube defects. An estimated 214,000–322,000 pregnancies globally are impacted by NTDs each year, with an estimated two instances per 1000 babies being the typical global prevalence of the condition. In lower income countries, prevalence and the negative consequences it causes are disproportionately high.^8^

Evaluating the socioeconomic and maternal characteristics that influence the occurrence of congenital anomalies among multiple ethnicities will provide more regional context. Due to limited literature addressing these risk factors in people of different ethnic groups, this study aimed to determine their association with congenital malformations in the socially diverse setting of Karachi.

## METHODS

This multi-center, case-control study spanned over a duration of six months (December 2022 – May 2023) included women ranging from 18–45 years using purposive sampling technique. It was conducted at Jinnah Postgraduate Medical Center and Tanveer Ultrasound Clinic. Pregnant women visiting the radiology department were approached and for each participant the study parameters were filled out on a subject evaluation form. Sample size was estimated to be 120 (60 cases and 60 controls), calculated by using population prevalence and the open-source calculator www.openepi.com, version 3.01-SSPropor.

### Ethical Approval:

All procedures performed have been approved by the Ethical Review Committee of Bahria University Health Sciences campus (ERC 110/2022) and JPMC Institutional Review Board Committee (NO. F.2-81/2022-GENL/323/JPMC). Study parameters and objectives were explained, and participants were recruited after obtaining an informed, understood and voluntary consent.

### Inclusion & Exclusion Criteria:

Pregnant women between the ages of 18- 45 years were part of the study. Cases included pregnant women carrying fetuses with congenital malformations while pregnant women carrying fetuses without any congenital malformations were part of the control group. Pregnant women with gestational age less than 11 weeks, women with missed abortion, women with intrauterine death of the fetus and pregnant women who were not Balochi, Pashtun, Punjabi, Sindhi or Urdu-speaking were excluded from the study design.

### Imaging Technique:

Scans were obtained using Sonoscape S22 ultrasound system and Toshiba Aplio 500 ultrasound system, and a convex transducer with a frequency range of 1-7 MHz.

### Statistical Analysis:

Statistical package for social sciences (SPSS) version 23.0 was used for data analysis. After generating frequencies, Fischer’s exact and chi-squared tests were used to evaluate the association between maternal sociodemographic and risk factors with congenital anomalies. Binary logistic regression was applied to the observe the strength of association between variables. Results were considered significant if the p-value was ≤0.05 and highly significant if p-value was ≤ 0.01.

## RESULTS

Age ranged from 18-39 years. Out of the 60 cases, 43.3% belonged to 30-34 years’ age group while 45% of controls were in 25–29-year age group. Highly significant results were seen when association of maternal age and education level were tested using chi-square, suggesting that both variables are strongly associated with the risk of congenital malformations as shown in Table-I.

**Table-I:** Association of maternal sociodemographic factors with congenital malformations

Maternal Sociodemographic Factors	Cases n (60)	Controls n (60)	p-value	Binary Logistic Regression	

				OR	p-value
***Age***				
< 20	1 (1.7%)	3 (5%)	0.003**^ϵ^	7.800 [0.649 – 93.807]	0.106
20 – 24	11(18.3%)	8 (13.3)		1.891[0.478 – 7.486]	0.364
25 – 29	9 (15%)	27 (45%)		7.800 [2.173 – 27.993]	0.002**
30 – 34	26 (43.3%)	17(28.3%)		1.700 [0.513 –5.638]	0.386
35 – 39^R^	13 (21.7%)	5 (8.3%)		1	1
***Educational Level***					
Primary	23 (38.3%)	6 (10%)	0.000**^ϵ^	1.435 [0.248 – 8.291]	0.687
Secondary	22 (36.7%)	46 (76.7%)		11.500 [2.345 – 56.393]	0.003**
Higher Secondary	4 (6.7%)	6 (10%)		0.643[0.172 – 2.405]	0.512
Graduation^R^	11(18.3%)	2 (3.3%)		1	1
***Family Income***					
50,000 – 99,999	46 (76.7%)	55 (91.70%)	0.024*^ϵ^	3.348 [1.122 – 9.994]	0.030*
≥100,000 – 200,000^R^	14 (23.3%)	5 (8.3%)		1	1

p – value ≤ 0.05: statistically significant (*), p – value ≤ 0.01: highly statistically significant (**), Test applied = ^ϵ^ Chi-square, Binary logistic regression; OR: Odds ratio, CI: Confidence interval, R: Reference category, 1: Reference.

About 76.7% cases and 91.7% controls were from lower income group of 50,000 – 99,999 Pakistani rupees (PKR). On applying chi-square, it was observed that the lower income group showed significant results (p-value = 0.024) as compared to middle income group (≥100,00 - 200,000) comprising of 23.3% cases and 8.3% controls. Comparative assessment through binary logistic regression showed significant results for the lower income group (OR = 3.348, p-value = 0.03), (Table-I).

The BMI of cases as well as controls was within the normal range, leading to an insignificant result (0.128). Only one case belonged to >30 BMI. Use of medication was higher among the cases during the 1^st^ trimester giving a highly significant result (p < 0.000). Only one participant among the cases was exposed to rubella. Most of the participants including both cases and control used folic acid during the pregnancy showing an insignificant result (p = 0.088). Antenatal supplements were also used by majority of the cases as well controls (p = 0.272) as shown in Table-II.

**Table-II:** Association of maternal risk factors with Congenital Malformations

Maternal Risk Factors	Cases	Controls	p-value

	n	n	
***BMI***			
< 18	4 (6.7%)	4 (6.7%)	0.128^§^
18.5 – 24.9	37 (61.7%)	47 (78.3%)	
25 – 29.9	18 (30%)	9 (15%)	
>30	1 (1.7%)	0 (0%)
***Use of Medications***			
1^st^ Trimester	27 (45%)	2 (3.3%)	0.000**^§^
2^nd^ Trimester	4 (6%)	30 (50%)	
3^rd^ Trimester	4 (6%)	0 (0%)	
None	25 (41.7%)	28 (46.7)
***Exposure to Rubella***			
Yes	1 (1.7%)	0 (0%)	0.088^§^
No	26 (43.3%)	17 (28.3%)	
Did not get tested	33 (55%)	43 (71.7%)
Use of Folic Acid			
Yes	37 (61.7%)	43 (71.7%)	0.245^ϵ^
No	23 (38.3%)	17 (28.3%)
***Use of Antenatal supplements***			
Yes	54 (90%)	58 (96.7%)	0.272**^§^**
No	6 (10%)	2 (3.3%)	

p – value ≤ 0.05: statistically significant (*), p – value ≤ 0.01: highly statistically significant (**) Test applied =

ϵ Chi-square,

§ Fisher’s exact test.

**Fig.1:**
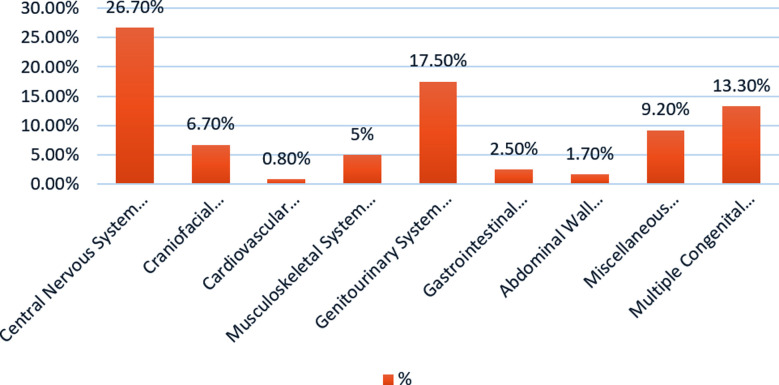
Distribution of Congenital Malformations among cases.

The distribution of various congenital malformations is shown in Fig.1. Anomalies of central nervous system were most frequent (26.7%), followed by craniofacial malformations (6.7%). Cardiovascular malformations were lowest (0.8%).Fig.1.

Sindhi, Punjabi, Balochi, Pashtun, and Urdu speaking ethnicities were evaluated. Significant results (p-value = 0.016) were obtained only for Urdu speaking ethnicity which exhibited a dominance of central nervous system (CNS) malformations on application of Fischer’s exact test as demonstrated in Table-III.

**Table-III:** Association of maternal ethnicity with Congenital Malformations

Congenital Malformations by organ system	Balochi	Pashtun	Punjabi	Sindhi	Urdu Speaking
***p-value***					
Central nervous system	0.169	0.862	0.408	0.690	0.016*
Craniofacial	0.255	1.000	0.458	0.121	0.062
Cardiovascular	1.000	1.000	0.192	1.000	1.000
Musculoskeletal	0.746	0.232	0.729	1.000	0.345
Genitourinary	0.634	0.393	0.072	1.000	0.801
Gastrointestinal	0.231	0.088	1.000	1.000	1.000
Body wall defects	0.361	0.388	1.000	1.000	1.000
Miscellaneous	0.298	0.323	0.914	0.712	0.924

p – value ≤ 0.05: statistically significant (*), p – value ≤ 0.01: highly statistically significant (**) Test applied = ^§^ Fisher’s exact test.

**Fig.2:**
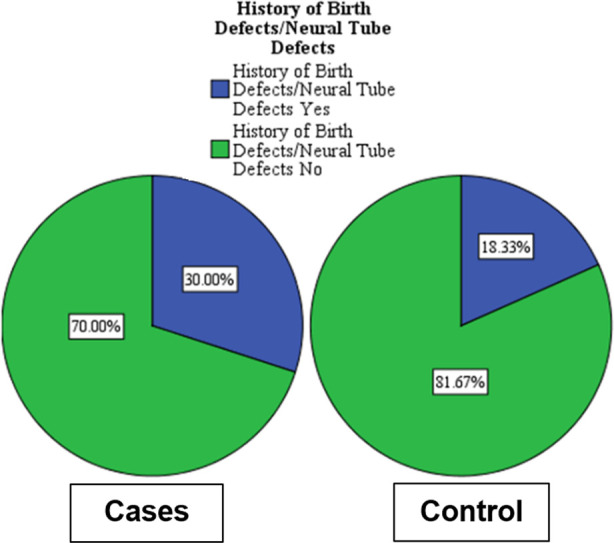
Distribution of Congenital Malformations with History of Birth Defects (Neural Tube Defects).

There was an increase when previous history of birth defects was acquired from the cases (30%) as compared to controls (18.33%) as shown in Fig.2. However, non-significant results were noted when the Chi square test was applied.

## DISCUSSION

We discovered an association between the incidence of CM and sociodemographic characteristics such as mother’s age, level of education, and family income. An increasing number of birth abnormalities are being addressed in clinical care and public health since the average age of pregnancy is higher now than it was in the past.^9^ CM can arise from chromosomal abnormalities, single gene mutations, and environmental influences. On the other hand, many anomalies have unknown causes.^10^

Advancing maternal age has been strongly implicated in chromosomal disorders such as aneuploidy. As age increases, so do meiotic abnormalities which leads to fetal anomalies.^11^ In our findings, although the cases belonged to a higher age group than the controls and there was an association, advanced maternal age was not a predictor for CM after adjusting for confounders. In Goetzinger’s study, maternal age over 35 years conferred a decreased risk of non-chromosomal abnormalities which was not observed in our study.^12^ Ahn et al. (2022) in their systemic review and meta analysis of observational studies found that maternal age ≥35 years was weakly associated with increased incidence of CM.^13^

Our study found a greater percentage of controls in the low socioeconomic class as compared to cases. Although the result was significant, it is likely attributed to a non-representative sample since more than three-fourth of the participants were from impoverished backgrounds and not all classes were adequately represented. However, a population-based study conducted in California by Pevandi et al.^14^ showed that it is challenging to separate the negative impacts of social disadvantage on health from the two primary determinants— environmental pollutants and socioeconomic position. They discovered that people with the lowest socioeconomic status are more likely to be exposed to hazardous contaminants which was associated with the development of congenital heart disease. Other studies have also established a relationship between socioeconomic disparity and the incidence of CM^13^, which may be explained by poor nutrition and restrictive access to healthcare resources. Similar results were seen in another study.^15^

Low maternal education frequently correlates with socioeconomic circumstances and is recognized as a risk factor for pregnancy-related problems and reduced adherence to guidance.^16^ Our study revealed a relationship between CM and lower educational attainment, as most of the cases attained primary level education, whereas most of the controls achieved secondary level education, suggesting that the mother’s personal choices— such as compliance with health-promoting practices, may have a larger influence than the socioeconomic environment.

Obesity (BMI > 30 kg/m^2^) is the leading, but preventable risk factor for poor pregnancy outcomes in many nations today. No significant association was found in the current investigation, however, Liaqat et al.^17^ reported that maternal obesity increases the likelihood of congenital anomalies, supporting the idea that there is an association between BMI and congenital malformations.

Usage of folic acid and other antenatal supplements did not correspond with the development of congenital malformations in the current study. This was likely because due to awareness programs, most of the pregnant women consume folic acid as well as other antenatal supplements in their diet. On the other hand, the use of other medications was shown to have a significant association. Antibiotics, anti-asthmatic, anti-epileptic, antihypertensive, antipsychotic, anti-thyroid, and renal treatments were the most regularly prescribed drugs in the current study. Additionally, several papers have demonstrated a significant relationship between drug usage and CM.^18^,^19^ In the current research, there was just one incidence of rubella exposure, and no statistically significant association was found. In contrast, Wang et al.^20^ and Gupta et al.^21^ found an established relationship between rubella exposure during the first trimester and congenital cardiac defects.

The current investigation revealed preponderance of CNS malformations among all congenital malformations. This was supported by another study in which neural tube defects were most common along with hydrocephalus among other co-existing CM.^22^

Our research revealed no significant association between birth defects and prior history of congenital anomalies. However, a retrospective and cross-sectional research conducted in 23 rural sub-centres of block Beri, district Jhajjar (Haryana, India) among 920 women, found that pregnant women with a history of congenital anomalies (8.3%) had a higher incidence of congenitally malformed newborns, with a 2.6 times greater chance of having a malformed baby. This might be attributed to the use of numerous settings and a larger sample size.^23^

A significant association was discovered between Urdu-speaking mothers and central nervous system (CNS) abnormalities in the current research, the findings cannot be generalized due to limited sample size. The cause of CNS anomalies can be attributed to both hereditary and environmental factors; however, no research has been conducted relating CNS anomalies to ethnic variations, so it cannot be compared to other studies. As a relatively new finding, it must be explored further with larger cohorts and may provide the groundwork for further genetic associations.

This study has provided a detailed insight of how congenital malformations differ across ethnicities, assisting in the identification of distinct patterns and occurrence rates. Healthcare practitioners could use this information to develop targeted screening and counselling programs for pregnant women from high-risk ethnic groups. Furthermore, policymakers may use this data to develop targeted public health programs to improve awareness about the importance of prenatal care and healthy mothering practices among specific ethnic groups.

### Limitations:

Time constraints did not allow for a more detailed study with follow-up of patients. Another limitation is that exposure to tobacco smoke was not reported.

## CONCLUSION

Variables that were associated with an increased incidence of congenital abnormalities included age group, education level, socioeconomic status, and medication use during the first trimester. Our study found a significant association between congenital abnormalities of the central nervous system and Urdu-speaking mothers as compared to Balochi, Pashtun, Punjabi, and Sindhi mothers. Nevertheless, because of the small sample size and time constraints, the results cannot be generalized to the entire population.

### Authors’ Contributions:

**JA** conceived, designed, manuscript drafting, data acquisition and analysis and interpretation.

**AQ** editing, critical review, accountability and integrity of work.

**AS** manuscript drafting, data analysis and interpretation and critical review.

**EA** Data analysis and interpretation, manuscript drafting and review.

All authors have read the final version and are also responsible for the integrity of the study.
